# Clinical Performance Comparison of a Long Versus a Short Axial Field-of-View PET/CT Using EARL-Compliant Reconstructions

**DOI:** 10.1007/s11307-024-01939-5

**Published:** 2024-08-02

**Authors:** Mostafa Roya, Johannes H. van Snick, Riemer H. J. A. Slart, Walter Noordzij, Gilles N. Stormezand, Antoon T. M. Willemsen, Ronald Boellaard, Andor W. J. M. Glaudemans, Charalampos Tsoumpas, Joyce van Sluis

**Affiliations:** 1 Department of Nuclear Medicine and Molecular Imaging, University of Groningen, University Medical Center Groningen, Hanzeplein 1, 9713 GZ Groningen, The Netherlands; 2 Department of Biomedical Photonic Imaging, Faculty of Science and Technology, University of Twente, Enchede, The Netherlands; 3 Department of Radiology and Nuclear Medicine, Free University of Amsterdam, University Medical Centers Amsterdam, De Boelelaan 1117, 1081 HV Amsterdam, The Netherlands

**Keywords:** Oncology, Long axial field-of-view, 2-deoxy-2-[^18^F]fluoro-D-glucose, FDG, PET/CT, Prospective study

## Abstract

**Purpose:**

To ensure comparable PET/CT image quality between or within centres, clinical inter-system performance comparisons following European Association of Nuclear Medicine Research Ltd. (EARL) guidelines is required. In this work the performance of the long axial field-of-view Biograph Vision Quadra is compared to its predecessor, the short axial field-of-view Biograph Vision.

**Procedures:**

To this aim, patients with suspected tumour lesions received a single weight-based (3 MBq/kg) 2-deoxy-2-[^18^F]fluoro-D-glucose injection and underwent routine clinical ($\sim$ 15 min) scans on the Vision and 3-min scans on the Quadra in listmode in balanced order. Image quality (IQ), image noise (IN), and tumour demarcation (TD) were assessed visually by four nuclear medicine physicians using a 5-point Likert scale and semiquantitative analysis was performed using standardised uptake values (SUVs). Inter-reader agreement was tested using Wilcoxon’s signed rank test and the SUVs were statistically compared using a paired t-test.

**Results:**

Twenty patients (mean age, 60 years ± 8.8 [standard deviation], 16 male) were enrolled. Inter-reader agreement ranged from good to very good for IQ and IN (0.62 ≤ W ≤ 0.81), and fair for TD (0.29 ≤ W ≤ 0.39). Furthermore, a significant difference was found for TD (*p* = 0.015) between the systems, showing improved TD for the Quadra.

**Conclusion:**

This study demonstrates that the Quadra can be used in routine clinical practice with multiple PET/CT systems or in multicentre studies. This system provides comparable diagnostic image quality and semiquantitative accuracy, improved TD, and has the advantage of shorter scan durations.

## Introduction

In recent years, technological innovations have spurred the use of diagnostic PET/CT in the clinical practice. Conventional systems now have, for example, silicon photomultipliers (SiPM), resolution modelling, and time-of-flight application (TOF) [1–5]. In 2018, the SiPM-based Biograph Vision PET/CT (hereinafter referred to as Vision (Siemens Healthineers)) produced images with superior quality and lesion detectability compared to conventional systems based on photomultiplier tubes [6, 7].

More recently, a major development in PET imaging has been the extension of the axial field-of-view (AFOV). Studies have shown that a larger AFOV results in higher photon detection sensitivity, thereby enhancing image quality and lesion detectability [8]. The increased sensitivity has resulted in substantially improved image quality, enabling optimised protocols and new clinical applications [8–15]. For example, scan time reduction of PET examinations are expected to lead to high patient throughput, paediatric protocols without general anaesthesia, scanning ICU patients, and increased patient comfort, especially for claustrophobic patients [9]. Despite the expectation that scanning claustrophobic patients would be more difficult due to the longer AFOV, our experience shows that these patients have a clear preference of being scanned on the LAFOV because they prefer a much shorter acquisition. Additionally, instead of reducing the scan time, the amount of activity administered to the patients can be reduced, resulting in new clinical applications still. Some primary examples are imaging radiosensitive patient populations (children, pregnant women), using radiotracers with poor labelling efficiency, or scanning the same patient multiple times or at later time points for disease activity monitoring [9].

The Biograph Vision Quadra (hereinafter referred to as Quadra (Siemens Healthineers)), a long AFOV (LAFOV) PET/CT consists of four detector electronic assembly rings in a row, versus the one that the Vision has, thereby extending the AFOV from 26.3 cm to 106 cm [16]. The superior performance of the Quadra compared to conventional PET/CT systems has already been established [8, 9, 16–21], however, studies on the practical implications for the clinic are scarce [10–16, 19, 22]. Alberts et al. have shown that in a head-to-head comparison between the Biograph Vision 600 and Biograph Vision Quadra, the latter has improved subjective image quality, lesion quantification, and signal-to-noise ratio (SNR) [8]. In this study they estimated the duration of a Quadra scan that would give similar count statistics and equivalent image metrics as a Vision scan of 106 cm, with a bed velocity of 1.1 mm/s (total scan time of 16 min). This was shown to be 1.8 ± 0.85 min (mean ± SEM) for subjective image quality, 1.63 ± 0.19 min (mean ± SEM) for integral lesion activity, and 1.82 ± 1.00 min (mean ± standard error) for SNR, with an injection of 3.5 MBq/kg 2-deoxy-2-[^18^F]fluoro-D-glucose ([^18^F]FDG) [8]. In this analysis of the scan times for the Quadra with equivalent count statistics as routine clinical scans on the Vision, however, no comparison was performed between the two PET/CT systems for reconstructions according to the guidelines provided by the European Association of Nuclear Medicine (EANM) Research Ltd. (EARL) [23]. These guidelines aid in harmonisation and standardisation of intersystem and inter-institutional tumour imaging.

The aim of this study was to make a semiquantitative and qualitative comparison of the clinical performance of the short AFOV (SAFOV) and LAFOV PET/CT using the reconstruction protocols provided by EARL. In this comparison of image quality (IQ), image noise (IN), and tumour demarcation (TD), routine clinical protocols on the SAFOV were compared to scan protocols on the LAFOV of only 3 min.

## Materials and Methods

### Patient Population

The local medical ethics review board of the University Medical Center Groningen waived the need for a formal ethical review (waiver number METc2020/554) of the study protocol. Patients referred for a diagnostic oncological PET/CT protocol between 9 and 23 September 2021 were consecutively requested to participate in this prospective, non-randomised clinical trial. Patients were informed regarding the aims, study procedures, and additional radiation exposure resulting from the extra low-dose CT (~ 1 mSv) and gave written consent to participate. Patients with suspected lesions were included, no distinction was made with regards to the type or severity of the disease. Patients with (possible) pregnancy and/or glucose levels above 11 mmol/L were excluded. Preparation and execution of the protocols were performed according to the EANM PET/CT tumour imaging guidelines [23, 24]. All patients received a weight-based injection of 3 MBq/kg of [^18^F]FDG and were scanned 60 min post-injection. In addition to the primary scan, a secondary scan was performed directly after the first one without an extra injection of radioactivity. This study was performed in a cross-over design, i.e., ten patients were scanned first on the SAFOV system and then on the LAFOV system, whereas the second ten patients were scanned in the reverse order. The time interval between the first and second scan for the SAFOV Vision-first (VF) and LAFOV Quadra-first (QF) groups was 31 ± 6.3 min (mean ± SD) and 18.4 ± 2.92 min (mean ± SD), respectively.

### Acquisition and Reconstruction Protocols

On the SAFOV Vision system, acquisition was done in a step-and-shoot mode of 120 or 180 s per bed position depending on the body weight of the patient, as per local clinical protocol. The patients were scanned from vertex to thighs and depending on patient length, the number of bed positions was between 6 and 9. On the LAFOV Quadra system, the patients were scanned with a static scan of one bed position for 3 min in listmode. This duration was chosen in order to account for the sensitivity difference between the scanners of a factor of 5 (16.4 cps/kBq vs 83 cps/kBq) [7, 16]. In our center, patients are generally scanned for 15 min on average on the SAFOV Vision, therefore the duration of the acquisition on the LAFOV Quadra was chosen to be 3 min. Additionally, this shorter scan duration was chosen in order to match the scan duration found by Alberts et al. as closely as possible while being conservative in image quality degradation for the sake of clinical image reading [8]. Also, Alberts and colleagues found scan duration of around 2 min, however the injected activity was slightly higher than in the current study (3.5 MBq/kg vs. 3 MBq/kg). Further, all images were reconstructed using a maximum ring difference (MRD) of 85, which was the only available option at the time of data collection. This setting corresponds to a photon acceptance angle of 18 degrees. For both systems, a low-dose CT scan (with an X-ray reference tube current of 30 mAs, a reference tube voltage of 100 kV, and a spiral pitch factor of 1) was acquired for attenuation correction. Subsequently, qualitative and semiquantitative analysis was performed on three different image reconstructions. First, the vendor-recommended reconstruction protocol was used, which is optimised for clinical image reading, hereinafter referred to as ‘CLIN’. Second, the two protocols with EARL-compliant settings will hereinafter be referred to as EARL 1 and EARL 2 [23]. More details regarding the reconstruction parameters are shown in Table 1. In order to meet the EARL criteria, post-reconstruction blurring filters were used for the EARL reconstructions. The CLIN protocol does not use any post-reconstruction filters to avoid degrading the image resolution. All reconstructions were performed using the ordered subset expectation maximisation with TOF. Resolution modelling was also applied as described by Panin et al. [25, 26].

**Table 1:** Different reconstruction protocols that were used for the comparison between the LAFOV Quadra and SAFOV Vision. CLIN refers to local settings that are used for clinical diagnosis. EARL 1 & 2 are reconstruction protocols provided by European Association of Nuclear Medicine Research Ltd. (EARL) for harmonisation of nuclear medicine data

Parameter	CLIN	EARL 1	EARL 2
Voxel size (mm^3^)	1.6 × 1.6 × 1.5	3.3 × 3.3 × 1.5	3.3 × 3.3 × 1.5
Image matrix size	440 × 440 × 708^a^	220 × 220 × 708^a^	220 × 220 × 708^a^
Gaussian filter kernel	None	7 mm	5 mm
Iterations/subsets	3/5	4/5	4/5

^a^The images obtained on the Quadra had 708 slices for matching the number of CT slices. The number of slices for the images obtained on the Vision varied with patient length

### Qualitative Assessment

All reconstructed images were rated visually by four nuclear medicine physicians: RS, AG, WN, and GS, with 25, 15, 15, and 11 years of experience in [^18^F]FDG image reading, respectively. The rating was done using a 5-point Likert scale for overall image quality, image noise, and tumour lesion demarcation (e.g. 1 = very poor, 2 = poor, 3 = neutral, 4 = good, 5 = very good) [6]. The rating physicians were blinded to the respective PET/CT system and received the images randomly. All reconstructed images were analysed using a dedicated Syngo.via VB40 workstation (Siemens Healthineers).

### Semiquantitative Assessment

Standardised uptake values (SUV) of lesions and healthy tissues were measured using the ACCURATE software tool [27]. SUV_mean_ was measured by placing spherical volumes of interest (VOI) of 3 cm diameter in the liver and lung and 1.5 cm in the ascending aorta (AA) for measuring the blood pool activity. Lesions were delineated with a semi-automated algorithm (A50P) and SUV_max_ and SUV_peak_ were measured [28]. This adaptive method calculates the isocontour of the 50% SUV_peak_ (1 ml region with the highest mean value inside the VOI) of the lesion with a correction for the background. SUV_mean_ (mean value of all voxels in the VOI) values of healthy tissue and SUV_max_ (maximum value in VOI) and SUV_peak_ (hottest 1 cm^3^ region in VOI) values of lesions were compared between the systems.

### Statistical Analysis

Statistical analysis was performed using the R programming language (version 4.2.2). Inter-observer agreement was assessed using Kendall’s W test of concordance. Normality was assessed visually using QQ-plots, and in cases of deviation, the Kolmogorov–Smirnov test was used to test if the differences were significant. The Wilcoxon’s signed rank test was used to test for significant differences in visual assessment, and the two-sided paired student’s t-test was used for the semiquantitative measurements. Lastly, Bland–Altman plots were analysed for the inter-system agreement analysis.

## Results

### Patient Population

Overall, 25 patients gave their consent to participate in the study. Five participants were excluded due to a high blood glucose level (> 11 mmol/L); not showing up for the scans; too much pain in order to continue the second scan; and two patients withdrew after the first scan. In total, 20 patients (16 male, 4 female) between 46 and 72 years were included (mean ± SD: 60 ± 8.8 years). The baseline demographics and clinical data of the patients are shown in Tables 2 and 3. The patients received between 160 and 325 MBq (mean ± SD: 251.6 ± 46.1 MBq) of FDG. The blood glucose levels were between 4.8 and 6.9 mmol/L (mean ± SD: 5.6 ± 0.5 mmol/L). The scan duration for the clinical protocol on the SAFOV system was between 12.5 and 21.6 min (mean ± SD: 15.5 ± 3.1 min). As a result of the shorter scan duration on the LAFOV system, the time difference between the first and second scans was significantly different in the VF and QF groups (mean ± SD: 31 ± 6.3 min, mean ± SD: 18.4 ± 2.92 min respectively, *p* < 0.05). The time difference between the injection and the second scan was also significantly different (*p* < 0.0001). For the VF group, the time difference between the injection and the two scans was 60 ± 5 min (mean ± SD, range: 55 to 69 min) (first scan) and 92 ± 7 min (mean ± SD, range: 77 to 100 min) (second scan). For the QF group this was 60 ± 2 min (mean ± SD, range: 57 to 64 min) (first scan) and 78 ± 3 min (mean ± SD, range: 75 to 84 min). For these two groups, the baseline demographics and clinical data are shown in Table 3. In order to be able to generalise the results, no distinction was made in the type or severity of cancer.

**Table 2:** Characteristics of all the patients that were included. No distinction was made in the type of disease and patients received a weight-based injection of 3 MBq/kg of [^18^F]FDG

Patient no	Age (y)	Sex	Weight (kg)	Disease	Activity (MBq)
1	49	M	82	Hodgkin lymphoma	250
2	62	M	96	Lung cancer	280
3	48	M	52	Oropharyngeal cancer	160
4	67	V	67	Melanoma	210
5	46	V	84	Lung cancer	260
6	71	M	86	Esophageal cancer	260
7	62	M	100	Esophageal cancer	295
8	62	M	86	Sarcoidosis	265
9	71	M	89	Lung cancer	270
10	61	M	84	Non-Hodgkin lymphoma	250
11	62	M	53	Lung cancer	175
12	70	M	75	Lung cancer	230
13	69	M	100	Lung cancer	315
14	46	M	95	Lung cancer	295
15	46	M	76	Melanoma	230
16	59	M	108	Lung cancer	325
17	60	V	97	Breast cancer	290
18	50	M	95	Lung cancer	280
19	65	V	67	Breast cancer	200
20	59	M	57	Melanoma	190

**Table 3:** Demographics of the patients. Patients underwent sequentially a scan on both systems. The patients were divided into two equal groups of 10 patients that were first scanned either on the Vision (Vision First, VF) or the Quadra (Quadra First, QF)

Parameter	Total group (*N* = 20)		VF (*N* = 10)		QF (*N* = 10)	
	Mean (SD)	Range	Mean (SD)	Range	Mean (SD)	Range
Age (years)	60 (8.8)	46 – 72	61 (9.3)	46 -72	59 (8.7)	46 – 71
Injected Activity (MBq)	252 (46.1)	160 – 325	250 (38.7)	160 – 295	253 (54.6)	175 – 325
Blood Glucose (mmol/L)	5.6 (.5)	4.8 – 6.9	5.7 (.6)	4.8 – 6.9	5.5 (.4)	5.0 – 6.1
Body weight (kg)	82.5 (16.3)	52 – 108	82.6 (13.9)	52 – 100	82.3 (19.3)	53 – 108
Duration protocol SAFOV (s)	928 (183.5)	749—1298	950 (197.2)	751 – 1298	906 (197.2)	749 – 1298
Time between start scans (min)	24.7 (8.0)	15 – 40	31.0 (6.2)	21 – 40	18.4 (2.9)	15 – 24

### Qualitative Assessment

**Fig. 1:**
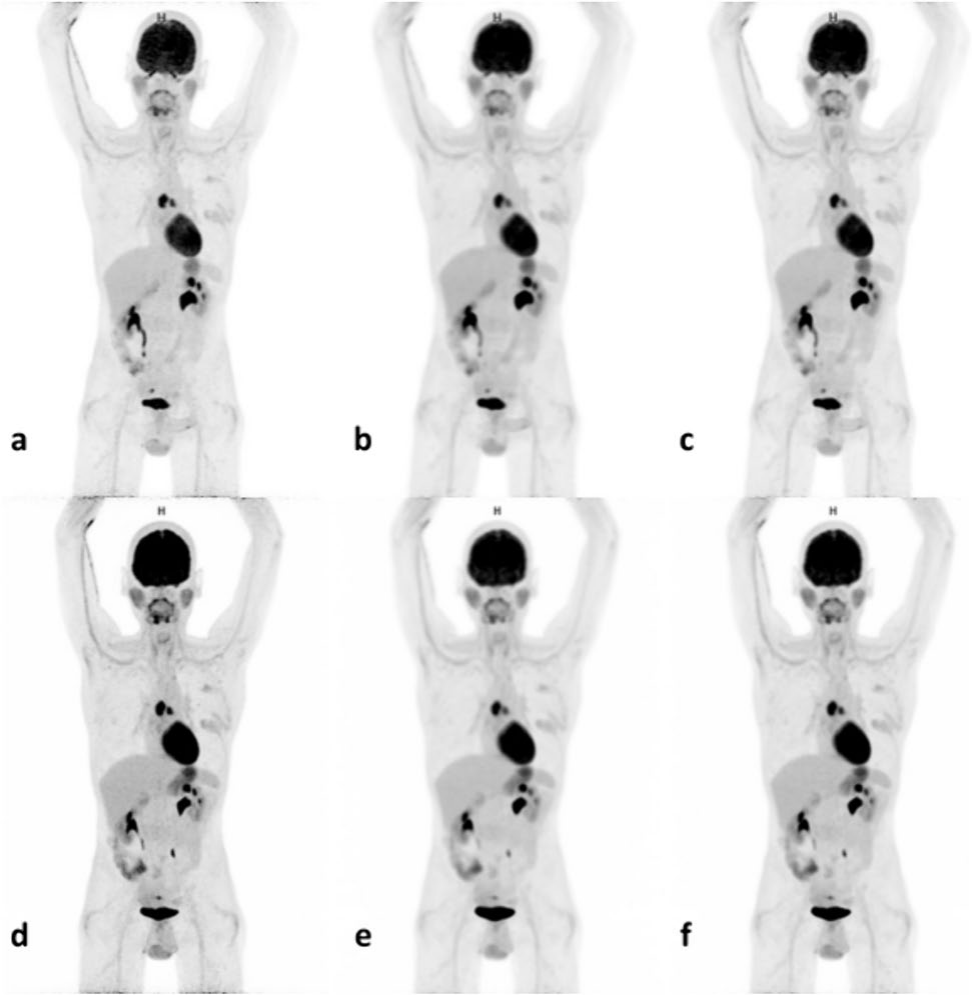
Maximum intensity projection images from CLIN (**a** & **d**), EARL 1 (**b** & **e**) and EARL 2 (**c** & **f**). Reconstructions obtained from the SAFOV system (top row) and LAFOV system (bottom row)

Kendall’s coefficient of concordance (W) showed moderate to high (0.62 > W > 0.81) agreement between the rating of the physicians for overall image quality and image noise (Table 4). However, the scores for tumour demarcation were low for both the Vision and the Quadra (W = 0.39 and W = 0.29, respectively). The Wilcoxon Signed Rank test showed that the nuclear medicine physicians gave significantly more often higher scores for TD when scans were performed on the LAFOV Quadra system (Z = -2.69, *p* = 0.015). No significant differences were found for overall image quality or image noise. The positive and negative ranks are shown in Table 4. Differently reconstructed images for patient number 3 are shown in Fig. 1.

**Table 4:** Results Wilcoxon Signed Rank test and Kendall's W

Comparison (Quadra versus Vision)	Ranks	Number	Percent	Kendall’s W	
				Vision	Quadra
Overall image quality	Positive	49	20%	0.63	0.62
	Equal	147	61%		
	Negative	44	18%		
	Total	240			
Image noise	Positive	36	15%	0.81	0.75
	Equal	175	73%		
	Negative	29	12%		
	Total	240			
Tumour demarcation	Positive	68	28%	0.39	0.29
	Equal	131	55%		
	Negative	41	17%		
	Total	240

### Semiquantitative Assessment

**Fig. 2:**
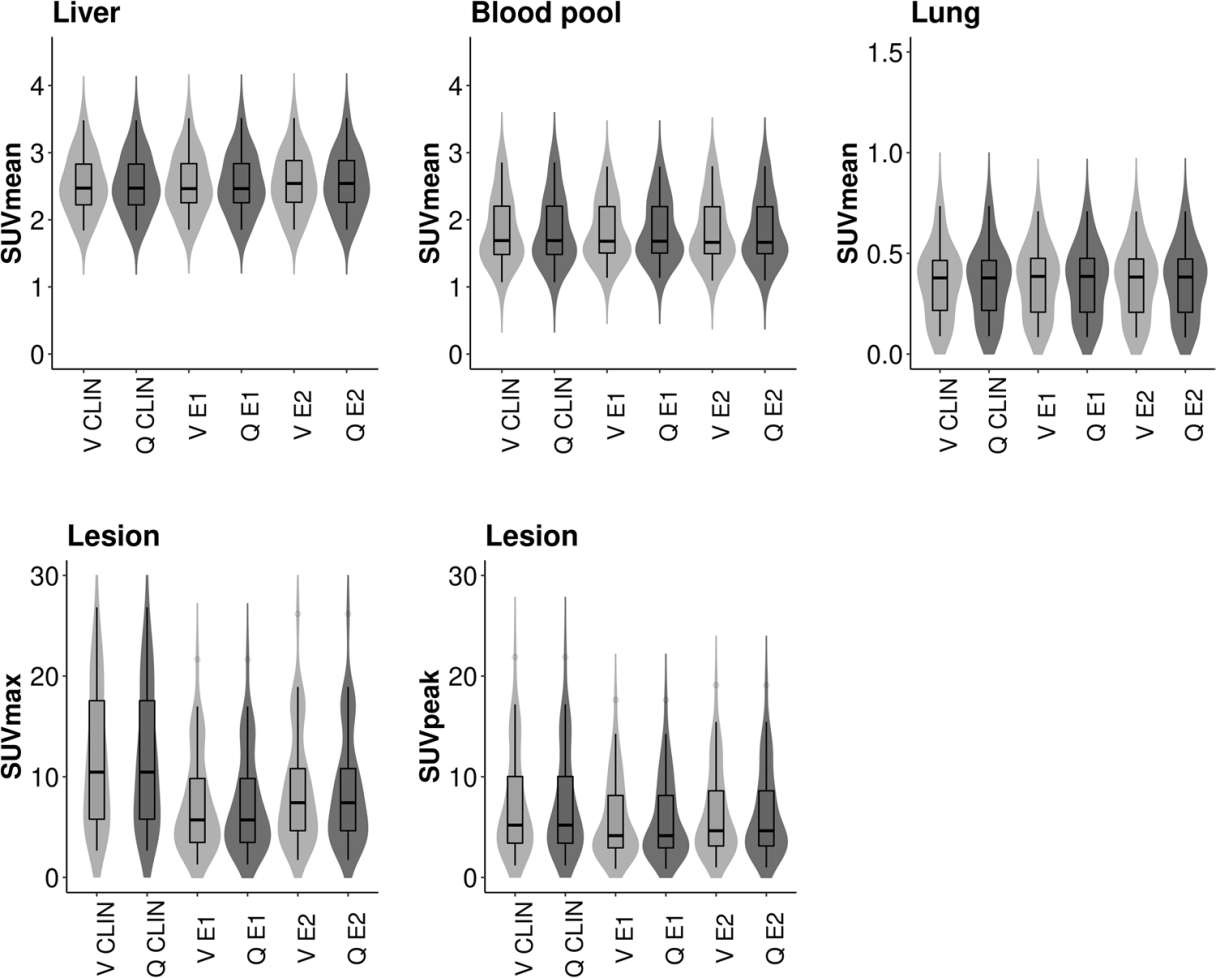
Boxplot of healthy tissue (SUV_mean_) and lesions (SUV_max_ & SUV_peak_). CLIN: local clinical reconstruction protocol; EARL 1 & 2: reconstruction protocols provided by the European guidelines (European Association of Nuclear Medicine Research Ltd.); V: Vision; Q: Quadra

**Fig. 3:**
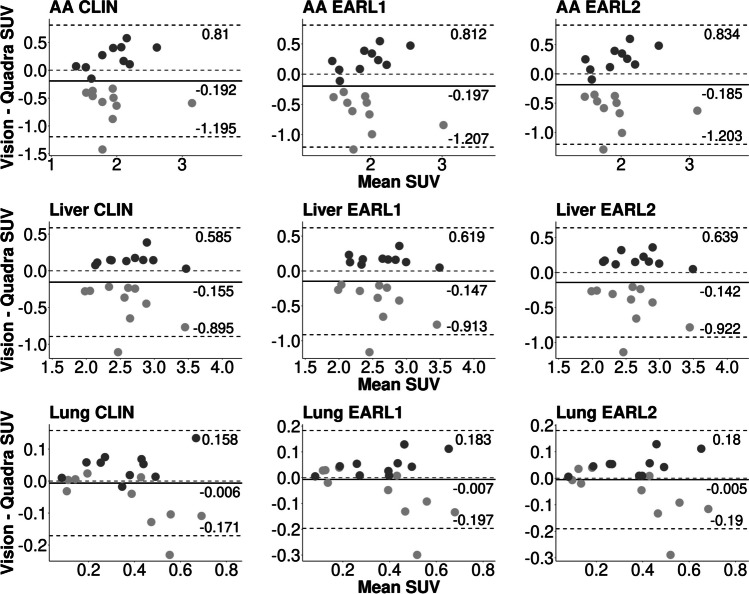
Bland–Altman analysis for intersystem comparison. SUV_mean_ values from the ascending aorta (AA), liver and lung tissue for the Vision-first (dark grey) and Quadra-first (light grey) groups. For each paired value, the difference is shown on the y-axis, and the average is shown on the x-axis. The lines of agreement are 1.96 * SD. CLIN: local clinical reconstruction protocol; EARL 1 & 2: reconstruction protocols provided by the European guidelines (European Association of Nuclear Medicine Research Ltd.); V: Vision; Q: Quadra

**Fig. 4:**
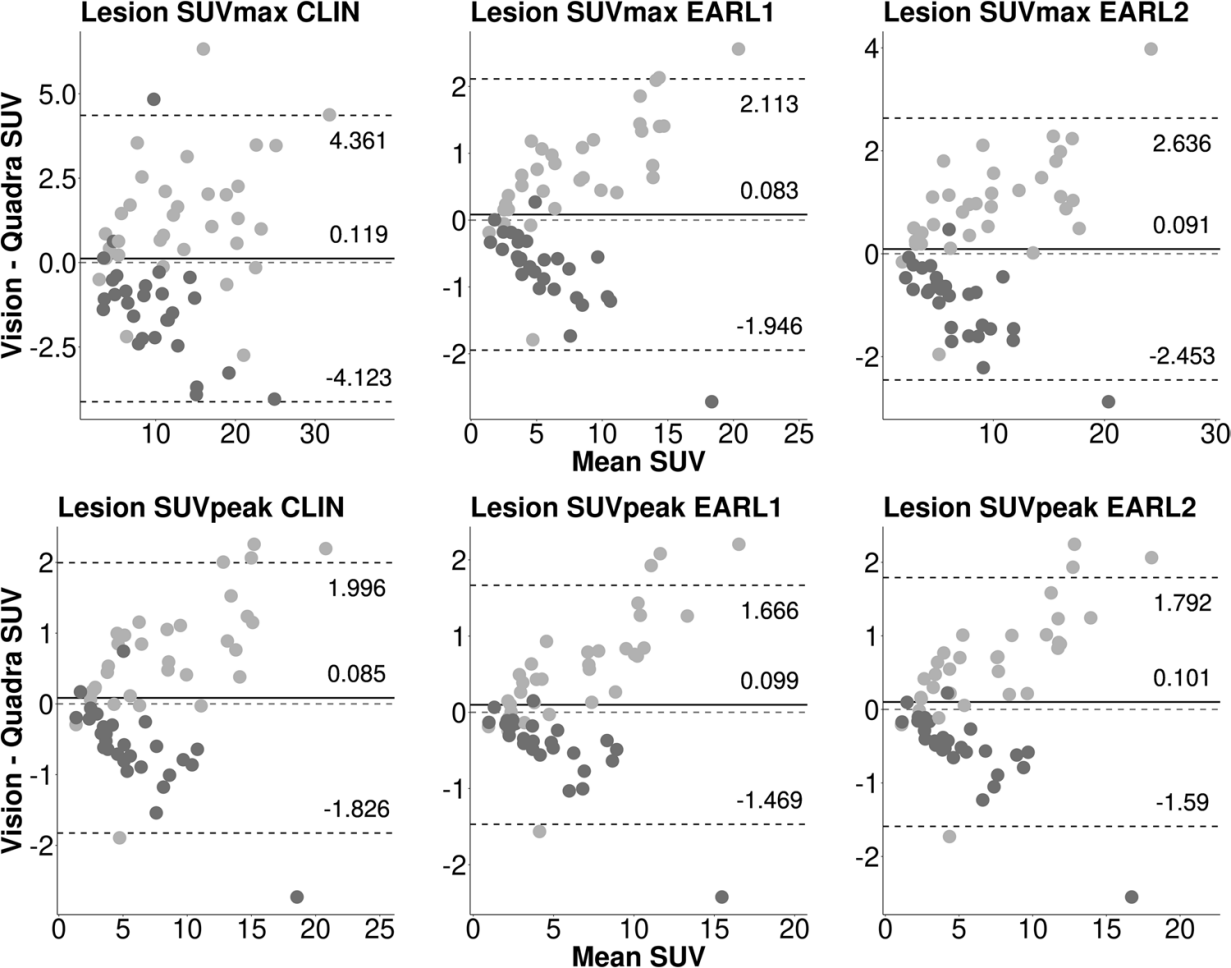
Bland–Altman analysis for intersystem analysis. SUV_max_ (top row) and SUV_peak_ (bottom row) of lesions for the Vision-first (dark grey) and Quadra-first (light grey) groups. Lines of agreement are set to 1.96 * SD. CLIN: local clinical reconstruction protocol; EARL 1 & 2: reconstruction protocols provided by the European guidelines (European Association of Nuclear Medicine Research Ltd.); V: Vision; Q: Quadra

The violin plots are shown in Fig. 2. The CLIN and EARL reconstructed images showed comparable quantitative metrics. The correlation for the SUVs between the two systems for lesion SUV_max_ and SUV_peak_ was high (0.90 < R^2^ < 0.96, Table 5). The correlation for SUV_mean_ for the lungs was moderate (0.77 < R^2^ < 0.83), and for the AA and the liver was low (AA: 0.13 < R^2^ < 0.18; liver: 0.38 < R^2^ < 0.42). Furthermore, the Bland–Altman plots show that between 90 and 96% of the measurements fall within the lines of agreement (Figs. 3 and 4).

**Table 5:** Results of two sample t-test & R^2^ values between the SUVs of the two systems with different reconstructions (CLIN: local clinical settings, EARL 1 & 2: protocols according to the guidelines of European Association of Nuclear Medicine Research Ltd. (EARL)). The p-value, t statistic of the t-test and the confidence interval (CI) are provided as well

	Mean SUV (SD)					
	Vision	Quadra	p-value	[CI]	t statistic	R^2^ (adjusted)
Blood pool (SUV_mean_) *N* = 20						
CLIN	1.84 (.50)	2.03 (.45)	0.21	[-0.50, 0.11]	-1.27	0.18 (0.14)
EARL 1	1.83 (.46)	2.03 (.45)	0.18	[-0.49, 0.09]	-1.37	0.13 (0.08)
EARL 2	1.83 (.49)	2.02 (.45)	0.22	[-0.49, 0.12]	-1.25	0.15 (0.10)
Liver (SUV_mean_) *N* = 20						
CLIN	2.53 (.44)	2.69 (.46)	0.28	[-0.44, 0.13]	-1.09	0.42 (0.39)
EARL 1	2.54 (.44)	2.69 (.47)	0.32	[-0.44, 0.15]	-1.02	0.41 (0.37)
EARL 2	2.55 (.44)	2.69 (.46)	0.33	[-0.43, 0.15]	-.99	0.38 (0.34)
Lungs (SUV_mean_) *N* = 20						
CLIN	0.36 (.18)	.36 (.20)	0.92	[-0.13, 0.12]	-.11	0.83 (0.82)
EARL 1	0.36 (.17)	.37 (.20)	0.91	[-0.13, 0.11]	-.11	0.77 (0.76)
EARL 2	0.36 (.18)	.36 (.20)	0.93	[-0.13, 0.12]	-.09	0.78 (0.77)
Lesion (SUV_max_) *N* = 61						
CLIN	11.97 (7.00)	11.85 (6.59)	0.92	[-2.32, 2.55]	.10	0.90 (0.90)
EARL 1	7.14 (4.65)	7.05 (4.28)	0.92	[-1.52, 1.69]	.10	0.95 (0.95)
EARL 2	8.53 (5.37)	8.44 (4.90)	0.92	[-1.75, 1.93]	.10	0.95 (0.94)
Lesion (SUV_peak_) *N* = 61						
CLIN	7.18 (4.78)	7.10 (4.44)	0.92	[-1.57, 1.74]	.10	0.96 (0.96)
EARL 1	5.65 (3.82)	5.55 (3.52)	0.88	[-1.22, 1.41]	.15	0.96 (0.96)
EARL 2	6.25 (4.17)	6.15 (3.87)	0.89	[-1.32, 1.54]	.14	0.96 (0.96)

## Discussion

In this clinical performance comparison between routine clinical protocols on the Vision and the 3-min acquisitions on the Quadra, qualitative analyses showed a significant difference for TD (Z = -2.69, *p* < 0.05). The images obtained from the LAFOV Quadra had a higher tumour to background ratio and received higher ratings for TD by all nuclear medicine physicians, meaning these images were visually perceived as better in terms of tumour margin delineation. The correlation between the SUVs obtained from both systems was lower in healthy tissue (0.13 < R^2^ < 0.46) compared to lesions (0.90 < R^2^ < 0.96), which can be attributed to a combination of the tracer kinetics and the sequence of acquisition and patient orientation inside the scanner. Due to the longer acquisition duration on the SAFOV Vision, the difference between the start of the first and the second scan is significantly different (Table 3, *p* < 0.05), which could affect the semiquantitative parameters [29, 30].

EARL 1 guidelines have been put in place to harmonise inter-system and multicentre study quantification [23]. Recently, the new EARL 2 standard was released, due to the emergence of new PET scanner technologies [31, 32]. The importance of the current study is that it does not only compare vendor-recommended reconstructions but also reconstructions according to the guidelines provided by EARL. All patient protocols were performed following EANM tumour imaging guidelines, such as patient preparation, acquisition, and reconstruction protocols [23, 24]. Therefore, this work indicates that the LAFOV Quadra can be used in harmony with other PET/CT systems in centres that have multiple scanners integrated into routine clinical practice or for multicentre research purposes.

Studies have shown that even with much faster scans on LAFOV, the resulting images are comparable to clinical scans on SAFOV. Pantel and colleagues demonstrated that the image quality of a 2-min acquisition on the PennPET Explorer (UPenn, KAGE Medical & Philips Healthcare) provided similar results as a 16-min acquisition on the 18 cm AFOV Ingenuity TF (Philips Healthcare) with an injected activity of approximately 8.8 MBq/kg FDG [33]. For the uEXPLORER (United Imaging Healthcare), a 194 cm AFOV PET/CT system, several studies have been published on the topic of scan time or administered activity reduction. To start with, Chen and colleagues showed in a paediatric study that even scans of 60 s on the 194 cm AFOV uEXPLORER (United Imaging Healthcare) could be sufficient for lesion detection with only 1.85 MBq/kg FDG [34]. After that, He et al. proposed a 2-min scan with only 1.85 MBq/kg of FDG in adult oncologic patients [35]. Further, Tan and colleagues found that scans of 8 min on the uEXPLORER were of sufficient diagnostic quality with only 0.37 MBq/kg of FDG [36]. Lastly, Hu and colleagues reported that 30 s to 45 s scans with 3.7 MBq/kg provided comparable image quality to a 13- to 16-min clinical scan on the uMI 780 (United Imaging Healthcare) with an AFOV of 30 cm [37]. The reduction in scan time for the current study is apparently smaller due to the fact that the SAFOV Vision is already a state-of-the-art PET/CT system with competitive features such as a TOF resolution of down to 210 ps [7]. In a comparative study, Alberts et al. showed that equivalent scan durations on the LAFOV Quadra for target lesion integral activity (mean ± SE: 1.6 ± 0.2 min), tumour-to-background ratio (mean ± SE: 1.8 ± 1.0 min), and qualitative analysis ratings (mean ± SE: 1.8 ± 0.9 min) were much shorter compared to the clinical scan on the Vision (16 min, 106 cm AFOV) [8]. Therefore, the results of the present study are in agreement with the results of the published studies previously mentioned.

The results of the current work demonstrated a significant difference in qualitative parameters between routine scans on SAFOV and 3-min acquisitions on LAFOV for TD (Z = -2.69, *p* < 0.05). The image quality of the resulting images obtained from the LAFOV Quadra was perceived as better by all nuclear medicine physicians. Compared to routine scan protocols (mean ± SD: 15.5 ± 3.1 min), the scan duration on the Quadra (mean ± SD: 3.0 ± 0.0 min) was reduced by more than a factor 5. This means that the Quadra can be used in clinics that include multiple PET/CT scanners or in multicentre research to provide comparable diagnostic (and semiquantitative) images, even when using shorter acquisition times, without compromising the diagnostic image quality of PET/CT.

However, some caveats are important to mention. One example is the overlap of bed positions on the SAFOV Vision due to the step-and-shoot setting of the acquisition, whereas the scans on the LAFOV Quadra were performed in a single bed position. Moreover, while the AFOV on the Quadra is currently fixed at 106 cm, the effective AFOV on the Vision varies with patient length (range: 919.5 mm, 1314.0 mm). This work compared the images with different scan durations without taking the effective AFOV into consideration. Furthermore, the current study was performed using a static bed position acquisition on the Quadra with a MRD of 85, which equals a photon acceptance angle of 18 degrees. At the time of the study, a maximum acceptance angle (MRD of 322) and continuous bed motion were not yet available for this system [38]. Increased photon acceptance angle (MRD 322) will further improve the sensitivity, yet a lower axial resolution was observed by Zhang and colleagues in the uExplorer [39]. Furthermore, no correction for motion was applied to the PET images. For the Quadra in particular, this type of correction is important since most of the body is in the field of view and any movement inside the AFOV could affect the resulting images.

In future studies, the influence of the maximum acceptance angle and continuous bed motion on the obtained image quality will be evaluated with regards to the photon scatter and the sensitivity profile of the camera. Second, the time between the first and second scans was statistically different between the groups, which could have affected the correlation between the semiquantitative parameters in healthy tissue (Table 3, *p* < 0.05) [29, 30]. Therefore, in upcoming trials, the period between the first and second scans will be kept as constant as possible. Lastly, the effect of motion correction on semiquantitative metrics needs to be investigated.

## Conclusion

This study shows that the new, fast LAFOV system, the Biograph Vision Quadra, has comparable clinical performance as the SAFOV PET/CT system, the Biograph Vision, and even significantly better tumour demarcation. The LAFOV Quadra system can be used in harmony in centres with multiple PET/CT systems or in multicentre research. Further, when using the LAFOV, scan duration or administered activity can be reduced without compromising the diagnostic power of PET/CT or compliance with the European tumour imaging guidelines (EARL 1 and 2). The current paper shows that a reduction of at least a factor of 5 can be achieved while providing comparable diagnostic images for routine clinical practice.

## Data Availability

The datasets that were used for this study are not publicly available because of sensitive information. However, the anonymised form can be made available on reasonable request.
